# Remission in asthma

**DOI:** 10.1097/MCP.0000000000001068

**Published:** 2024-03-05

**Authors:** Marek Lommatzsch

**Affiliations:** Department of Pneumology, University of Rostock, Rostock, Germany

**Keywords:** asthma, disease modification, remission

## Abstract

**Purpose of review:**

To review the current concepts of remission in asthma.

**Recent findings:**

Until 2023, asthma guidelines have been promoting the concept of disease control, recommending the step-wise addition of drugs until the best possible disease control is achieved. With the advent of highly effective, anti-inflammatory disease-modifying antiasthmatic drugs (DMAADs), treatment goals of asthma have changed. Several national guidelines have now announced remission as a general treatment goal in asthma. Currently, all guidelines agree that asthma remission is defined by the presence of at least three characteristics over a period of at least one 1 year: absence of exacerbations, no systemic corticosteroid use for the treatment of asthma and minimal asthma-related symptoms. In the future, a generally accepted, evidence-based and easy-to-use definition of remission will be needed for daily clinical practice. It is clear, however, that precise phenotyping (including measurement of biomarkers) is an essential prerequisite to achieve clinical remission in each individual patient.

**Summary:**

Remission has been included as the treatment goal in asthma in several national guidelines, reflecting the paradigm shift in asthma, from short-term symptom control to long-term symptom prevention. An international consensus on the criteria for asthma remission is expected in the near future.

## INTRODUCTION

Asthma treatment concepts have changed fundamentally over the last 100 years, from symptom treatment to symptom prevention [1^▪▪^]. This paradigm shift is associated with a change in the treatment goals of asthma, from asthma control to asthma remission. This article summarizes the evolution of the remission concept and current definitions of remission in asthma. 

**Box 1:**
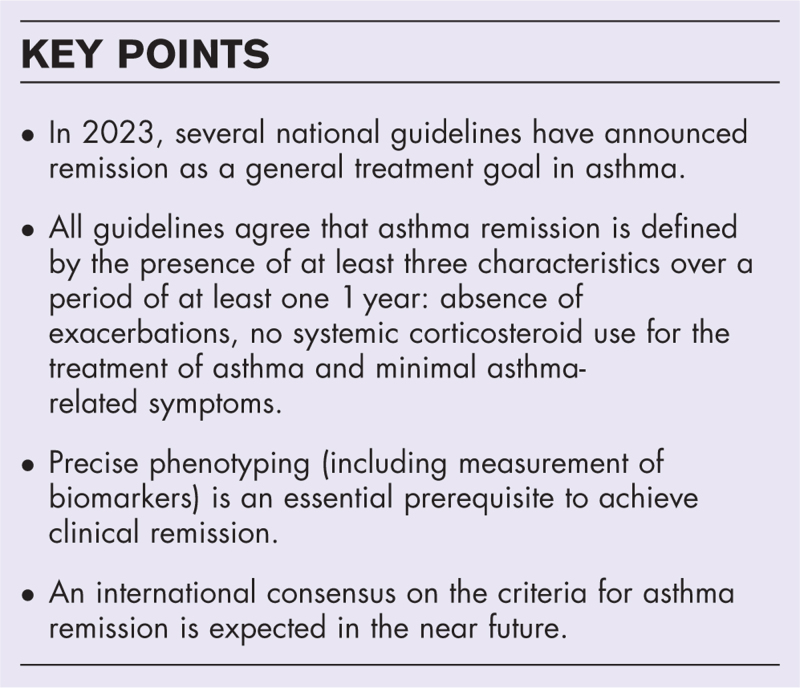
no caption available

## THE 20th CENTURY AND THE CONCEPT OF DISEASE CONTROL

Asthma therapy in the first half of 20th century was solely based on treatment with various symptom relievers, such as sympathomimetics, anticholinergics or methylxanthines [1^▪▪^]. The introduction of systemic glucocorticoids and short-acting beta-2 agonists (SABA) in the second half of the 20th century followed the same idea: the relief and control of recurrent symptoms. In addition, due to lacking alternative treatment options, it was generally accepted that these drugs can be associated with substantial adverse effects and increased mortality [1^▪▪^]. Furthermore, these drugs were not intended to modify the course or pathophysiology of the disease. Phenotyping of the patients was not mandatory in the 20th century: in all patients, it was recommended to add one drug to another using a standard step-up scheme (“treat-to-failure” approach) [2]. As a result, patients with severe disease were treated with multiple drugs in high doses, usually with substantial adverse effects. In line with this traditional concept (the step-wise addition of antiasthmatic drugs until the best possible disease control is achieved), all national and international asthma guidelines announced asthma control as the primary goal of asthma treatment [2].

## THE NEW WORLD OF ASTHMA TREATMENT OPTIONS

Over the last 50 years, there has been a huge progress in the understanding of the underlying pathophysiology of asthma. Although many cell types are involved in the pathogenesis of the disease, it has become clear that chronic airway inflammation with a type 2 signature is the major driver of asthma [3]. In addition, it has become apparent that there are several phenotypes of asthma (such as early-onset allergic asthma or adult-onset intrinsic asthma) with different and specific inflammatory profiles [4,5]. This scientific progress led to the development of highly effective anti-inflammatory drugs for the treatment of asthma [6]:

1. *Inhaled corticosteroids (ICS), either alone, or in combination with long-acting beta2-agonists (LABA) and long-acting muscarinic antagonists (LAMA).* ICS did not only revolutionize maintenance treatment, but also transformed on-demand asthma treatment. On-demand treatments with fixed combinations of ICS and fast-acting beta-2-agonists (ICS/FABA) are more effective and safer than on-demand treatments with SABA alone [7]: this led to the new concept of “Anti-Inflammatory Relievers (AIR)” in asthma [8]. Of note, oral leukotriene receptor antagonists (LTRAs) are less effective and have more potential adverse effects than ICS: therefore, LTRAs are currently not recommended as a first-line treatment option in asthma.
2. *Evidence-based allergen immunotherapy (AIT), either as sublingual (SLIT) or subcutaneous immunotherapy (SCIT)*. There is now an improved understanding of the underlying mechanisms of tolerance induced by AIT which can result in sustained disease modification of allergic asthma [9]. In addition, there is accumulating evidence from real-world studies that AIT can not only reduce exacerbations and improve symptoms in patients with asthma [10,11], but may also prevent the development of asthma [12].
3. *Biologics targeting specific inflammatory pathways in asthma*. There are currently six monoclonal antibodies from four different classes of biologics approved for the treatment of severe asthma [13]. These biologics are highly effective in reducing exacerbations and improving symptoms of patients with severe asthma, not only in clinical trials, but also in a growing number of large real-world studies [1^▪▪^].

These anti-inflammatory drugs were recently summarized under the umbrella term “disease-modifying antiasthmatic drugs” (DMAADs) [1^▪▪^]. The strength of DMAADs is not only their remarkable efficacy and safety, but also their potential collateral efficacy (beneficial effects on comorbidities) [1^▪▪^]. Thus, today there are highly effective and safe immunomodulatory drugs aiming at the prevention of symptoms by targeting the underlying type 2 inflammation, with the additional advantage of improving control of co-morbidities. However, it is clear that precise phenotyping of the patients is an essential prerequisite of any individually tailored DMAAD treatment [1^▪▪^].

## THE 21th CENTURY AND THE CONCEPT OF DISEASE MODIFICATION AND REMISSION

With the availability of highly effective DMAADs, the concept of asthma management has fundamentally changed: from the concept of disease control to the concept of disease modification [1^▪▪^]. The latter concept does not warrant the simple addition of one drug to another, but the identification of the right DMAAD(s) for the right patient [2]. The concept of disease modification with DMAADs is closely linked to the concept of asthma remission. Remission as a treatment goal, in conjunction with the treat-to-target approach, is well established in many other chronic inflammatory diseases such as rheumatoid arthritis [14,15] and inflammatory bowel diseases [16]. Of note, these remission concepts are based on the idea that remission can occur *on treatment*. In asthma, the term ‘remission‘ has previously been restricted to patients who became symptom-free either spontaneously (‘spontaneous remission‘) or remained symptom-free after completing a treatment (‘remission off treatment‘). However, the growing number of symptom-free patients with severe asthma while receiving biologic therapy in real life [17–19] has changed these views. As a consequence, new definitions of asthma remission are *independent* of the current treatment status, thus encompassing patients *on treatment*[1^▪▪^,^20^^▪▪^,^21^,^22^]. These remission definitions are also combined with a long-term vision (>12 months) [1^▪▪^,^20^^▪▪^,^21^,^22^], in contrast to a maximum of 1 month encompassed by the previous concept of asthma control. Of note: the term “remission” reflects a *disease state,* and should be distinguished from the term “super responder” which relates to a *treatment response*.

## NEW NATIONAL GUIDELINES: THE FIRST TO INCLUDE REMISSION AS A TREATMENT GOAL

As the first guidelines worldwide, the new German asthma guidelines for respiratory specialists, published in March 2023, have incorporated remission as the overarching asthma treatment goal [23^▪▪^]. The guidelines define the presence of *clinical remission* in asthma if four criteria are fulfilled for a period of at least 12 months: no exacerbations, no use of systemic steroids for the treatment of asthma, absence of asthma-related symptoms, stable lung function [23^▪▪^]. It is important to note that the criterion ‘no use of systemic steroids‘ refers to the treatment of asthma: treatment with systemic steroids for other conditions (e.g. adrenal insufficiency [24]) is not part of this criterion. In 2023, three other countries incorporated remission as the asthma treatment goal in their national guidelines: Spain, Japan and Italy (in all guidelines, a period of at least 12 months is mandatory to fulfill asthma remission criteria) [2]. The new Spanish guidelines (www.gemasma.com), published in May 2023, propose two types of remission: *clinical remission* (absence of asthma-related symptoms; no systemic steroids for the treatment of asthma; no exacerbations; optimization and stabilization of lung function) and c*omplete remission* (clinical remission plus absence of bronchial hyperresponsiveness and bronchial inflammation) [2]. In July 2023, the PGAM (Practical Guidelines for Asthma Management: for general practitioners in Japan) [25] defined *clinical remission* using the following criteria: an asthma control test (ACT) score ≥ 23 points, absence of exacerbations, and no use of systemic corticosteroids (a lung function criterion was not used in these guidelines) [2]. In August 2023, a severe Asthma Network Italy (SANI) Delphi consensus published two distinct clinical remission definitions (which will be incorporated in the upcoming new Italian asthma guidelines) [26]: *partial clinical remission* (no systemic steroids for the treatment of asthma and 2 out of 3 additional criteria: ACT score ≥20; no exacerbations; stable lung function) and *complete clinical remission* (no systemic steroids for the treatment of asthma; ACT score ≥20; no exacerbations; stable lung function) [2]. In September 2023, a consensus published by an American College of Allergy, Asthma, and Immunology (ACAAI), American Academy of Allergy, Asthma, and Immunology (AAAAI) and American Thoracic Society (ATS) workgroup [27] postulated six criteria for *clinical remission* in asthma (all six criteria are mandatory):

1. no exacerbations requiring physician visit, emergency care, hospitalization, and/or systemic steroid for asthma,
2. no missed work or school over a 12-month period due to asthma-related symptoms,
3. stable and optimized pulmonary function results on all occasions measured over a 12-month period with a minimum of two measurements during the year,
4. treatment with ICS only at low-medium doses (or less),
5. an ACT score >20 (or an AirQ score < 2 or an ACQ score < 0.75) on all occasions measured in the previous 12-month period with a minimum of two measurements during the year,
6. symptoms requiring one-time reliever therapy no more than once a month.

The discussion whether these complex and very ambitious clinical remission criteria are helpful to implement the concept of asthma remission into daily clinical practice is currently ongoing. However, regardless of the final consensus on the specific criteria of remission, it is clear that the concept of remission as the general treatment goal in asthma will become part of asthma guidelines worldwide. Of note, the Global Initiative for Asthma (GINA) will incorporate the concept of asthma remission in its documents in the near future.

## ROLE OF COMORBIDITIES

Comorbidities (such as obesity, diabetes, gastro-oesophageal reflux disease, chronic rhinosinusitis with or without nasal polyposis, anxiety and depression) – either associated with specific severe asthma phenotypes or induced by systemic corticosteroid treatment – need to be taken into account when assessing asthma symptoms [28^▪▪^]. Indeed, comorbidities such as obesity [29] have a significant negative impact on patient reported outcome measurements (PROMs) such as ACT and ACQ, implicating that so-called “asthma” symptoms might be overestimated and in fact be caused by comorbidities [30–32]. The number and severity of comorbidities in patients with asthma might also impact the optimal cut-off values of PROMs. Although an ACQ score of less than 0.75 might be an achievable goal in a patient with moderate-to-severe asthma without comorbidities, an ACQ score of even <1.50 might be difficult to reach in a patient with longstanding corticosteroid-dependent severe asthma and multiple corticosteroid-induced comorbidities (e.g. obesity, diabetes, osteoporosis and depression). Therefore, more research is needed to define the optimal PROMs and cut-off values to evaluate (the absence of) asthma symptoms in patients with asthma and comorbidities.

## CONCLUDING REMARKS

**FIGURE 1:**
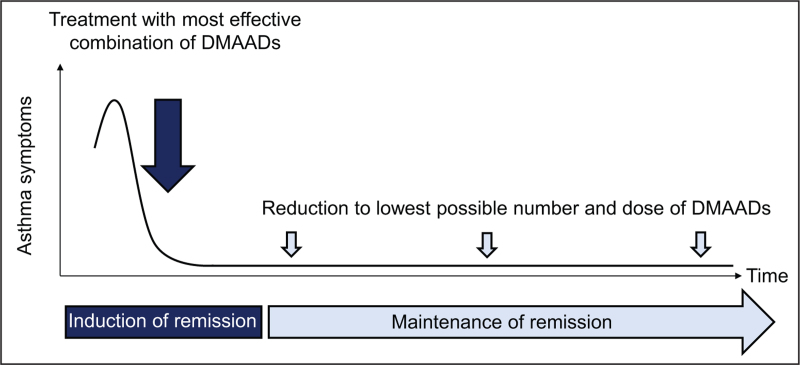
Asthma treatment concept in the future. DMAAD, disease-modifying antiasthmatic drug.

In the future, there will be a landscape of asthma treatment options in which DMAADs are individually combined with each other and used variably over time [8]. Instead of the traditional and uniform step-up escalation schemes, asthma treatment will consist of two phases: an initial phase of remission induction (treatment with the most effective individual combination of DMAADs) and a second phase of remission maintenance (using as few DMAADs as possible, in the lowest possible dose) (Fig. 1). Clearly, asthma remission is a continuum, the percentage of patients achieving remission increases or decreases depending on the criteria used. It is therefore mandatory to agree on a generally accepted definition of remission which has to ensure that only well controlled patients start reducing their treatment burden and that not too many patients are deprived of a remission. A realistic pathway to such a definition could be the systematic application of the currently used criteria to many clinical trials in severe asthma, extracting the characteristics of patients on long-term asthma control fulfilling the criteria of clinical remission. It is also important to establish to what extent control of underlying type-2 inflammation and airway pathology (i.e. biological remission, as suggested by the Spanish guidelines) is a component of remission. Studies such as the SHAMAL study [33^▪▪^] suggest that FeNO might be a relevant treatment target but more work is needed to confirm this.

## Acknowledgements

*None.*

### Financial support and sponsorship

*None.*

### Conflicts of interest

*M.L. reports grants for research or clinical trials, paid to his institution, from AstraZeneca, Deutsche Forschungsgemeinschaft (DFG), and GSK; and consulting fees, travel expenses, or honoraria for lectures from ALK, Allergopharma, AstraZeneca, Berlin-Chemie, Boehringer Ingelheim, Chiesi, GSK, HAL Allergy, Leti, Novartis, MSD, Sanofi, Stallergenes, and Teva.*

## REFERENCES AND RECOMMENDED READING

Papers of particular interest, published within the annual period of review, have been highlighted as:

▪ of special interest
▪▪ of outstanding interest
